# Myelination of the retinal nerve fibre layer: A case report

**DOI:** 10.1177/13524585251365793

**Published:** 2025-08-19

**Authors:** Gioia Riboni-Verri, Riddhima Gautam, Benson Chen, Nick G Cunniffe

**Affiliations:** Department of Clinical Neurosciences, University of Cambridge, Cambridge, UK; Cambridge Clinical Vision Laboratory, University of Cambridge, Cambridge, UK; Cambridge Clinical Vision Laboratory, University of Cambridge, Cambridge, UK; Department of Clinical Neurosciences, University of Cambridge, Cambridge, UK; Cambridge Clinical Vision Laboratory, University of Cambridge, Cambridge, UK; Department of Medicine, University of Auckland, Auckland, New Zealand; Department of Clinical Neurosciences, University of Cambridge, Cambridge, UK; Cambridge Clinical Vision Laboratory, University of Cambridge, Cambridge, UK

**Keywords:** Multiple sclerosis, myelin, remyelination, visual evoked potentials, neuroophthalmology

## Abstract

**Background:**

Aberrant myelination of the retinal nerve fibre layer (RNFL) can follow lamina cribrosa damage. Such cases may provide insight into how increased myelin affects the visual evoked potential (VEP), a key outcome in remyelination trials.

**Objective:**

We present the case of a myelinated RNFL in a patient with relapsing-remitting multiple sclerosis (RRMS) with accompanying electrophysiology.

**Methods:**

Visual assessments used a VisionSearch Plus, Heidelberg Spectralis optical coherence tomography, and Topcon fundus camera.

**Results:**

Full-field visual evoked potential (FF-VEP) peak time latency was abnormally short, 90 ms.

**Conclusion:**

This is the first known case report of a myelinated RNFL with electrophysiology, which supports the assertion that reductions in VEP latency correlate with myelination.

## Introduction

A myelinated retinal nerve fibre layer (RNFL) is an incidental anomaly characterised by the presence of ectopic myelin in the retinal nerve fibres anterior to the lamina cribrosa. This is typically benign but can be misinterpreted as one of several potentially serious conditions, such as cotton-wool spots, branch retinal artery occlusion, peripapillary epiretinal membrane, retinal pigment epithelium detachment, retinal infiltrates or retinoblastoma.^1^ The exact aetiology and pathophysiology of a myelinated RNFL remains largely unknown. In health, myelination of retinal ganglion cell (RGC) axons begins around 5 months of gestation at the optic tract, proceeding towards the globe, before terminating at the level of the lamina cribrosa.^2^ The current theory is that aberrant RNFL myelination occurs when oligodendrocyte progenitor cells (OPCs) either breach the lamina cribrosa before it fully matures or when a pathological insult transiently disrupts this barrier.^3^ This results in ectopic OPCs and the formation of a congenital, or acquired, myelinated RNFL.

Such cases may provide insight into the effects of OPC-mediated myelination on visual evoked potential (VEP) latency in humans. VEP latency is increasingly being deployed as a sensitive quantitative biomarker of remyelination in phase 2 remyelination trials.^4^ While demyelination is well established to prolong latency,^5–7^ evidence that reductions in latency directly reflect enhanced myelination remains limited in humans.^8^ Here, we report a case of a myelinated RNFL in a patient with relapsing-remitting MS (RRMS) with accompanying electrophysiology, showing an abnormally shortened VEP latency.

## Case presentation

We present the case of a 51-year-old gentleman with RRMS who attended a screening visit for the Cambride Centre for Myelin Repair trial Two (CCMR-Two) clinical trial of a possible remyelination-promoting combination of drugs (NCT05131828). Screening for this trial included a full-field VEP (FF-VEP) with a 2-Hz pattern-reversal stimulus of 60 arcmin check size (VisionSearch Plus system, Sydney, Australia). Averaged recordable signals were taken from a channel formed between gold-cup electrodes positioned frontally in the midline and 2.5 cm above the inion (Fz-Oz).^9^

**Figure 1.**
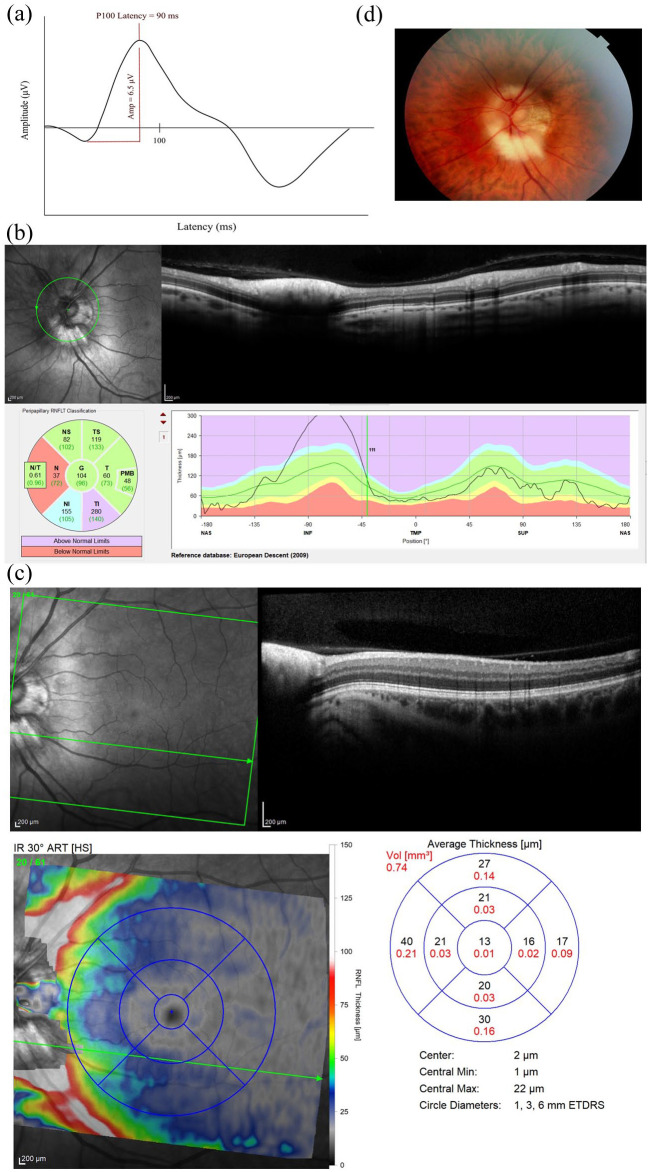
(a) Left-eye full-field visual evoked potential showing peak time latency of 90.0 ms. (b) Left-eye SD-OCT printout of the peripapillary RNFL. (c) Left-eye SD-OCT printout of the macula. (d) Left-eye fundus photography showing myelination of the peripapillary RNFL of the left optic disc. SD-OCT, spectral-domain optical coherence tomography; RNFL, retinal nerve fibre layer.

His MS had been treated with four cycles of alemtuzumab between 2003 and 2013, followed by an autologous haematopoietic stem-cell transplant 3 years previously following an episode of acute optic neuritis (ON) in the right eye. The right ON had resulted in a central scotoma which prevented fixation, and his FF-VEP was unrecordable. He had no history of left-sided ON but had previously been diagnosed with a myelinated RNFL (believed congenital) when he was diagnosed with MS following a brainstem relapse in 2000. No visual impairment was noted in his left eye. However his FF-VEP peak time latency was 90.0 ms (Figure 1(a)). While we could not directly compare this to his right eye, this value is more than 3 standard deviations (SD) below the calculated average from 64 healthy controls (111.2 ± 3.7 ms) and is the shortest VEP latency we have observed using this protocol across 178 MS participants screened for this trial. Given one of the selection criteria for trial eligibility in CCMR-Two was a significantly prolonged (⩾118 ms) FF-VEP latency in at least one eye, he was unable to proceed further with the study.

Upon further assessment of the left eye peripapillary retina and macular retina with spectral-domain optical coherence tomography (Figure 1(b)–(c)) (SD-OCT, Spectralis; Heidelberg Engineering; Heidelberg, Germany) and fundus photography (Figure 1(d)) (Topcon TRC-NW400 Non-mydriatic camera, Topcon Healthcare Solutions Incorporation; Tokyo, Japan), myelination of the peripapillary RNFL of the left optic disc was observed. This had a characteristically thickened and hyperreflective RNFL at the optic nerve head on SD-OCT. Visual field testing was not included in the trial-screening assessments. The most recent MRI brain imaging was performed 4 years prior: He had a moderate lesion load, with some lesions involving the optic radiations seen.

## Discussion

This case is, to our knowledge, the first description of a myelinated RNFL in a patient with MS with accompanying electrophysiology. In our case, myelination of the RNFL appeared confined to the peripapillary region, without macroscopic involvement of the macula or peripheral retina of the left eye. This was associated with an abnormally short FF-VEP latency, potentially highlighting the physiological effects of excessive myelination on neural conduction.

It has previously been shown *in vivo* that 1 mm of demyelination is associated with at least 1-ms VEP latency delay.^5^ We measured high density of myelin extending up to 3 mm from the disc (relative to the length of the RGC axon which can extend up to 50 mm).^10^ But this is likely to expand more peripherally, with lower density, and also does not include the non-visualised prelaminar optic nerve segment (from the surface of the optic disc to the lamina cribrosa) which approximates to 300 µm. It is unknown whether pathologically excessive myelination leads to a corresponding latency reduction, and it is unknown whether myelination behind the lamina cribrosa might have been similarly altered in this case. Of note, we still detected the reduced VEP latency, in the context of a handful of optic radiation lesions, which might have confoundingly lengthened the VEP latencies.

This case holds particular significance in the setting of clinical remyelination research.^4^ Currently, changes in VEP latency are viewed as the most sensitive way to measure remyelination in phase 2 trials.^8^ It has long been established that an increase in VEP latency follows demyelination,^11^ but only recently has it been confirmed (in animals) that the reduction in VEP latency following demyelination directly indicates remyelination, rather than ion channel redistribution, resolution of conduction block, or plasticity.^6,12^ Our observation of significantly shortened VEP latency in an eye with a myelinated RNFL supports the association between myelination and recovery of VEP latency and the validity of this outcome measure in human trials of remyelination-promoting drugs.

## Conclusion

This case strengthens the position that increased myelin in the visual pathway leads to quantifiable reductions in VEP latency in people. This is particularly important as it supports the use of VEP latency as an outcome measure in clinical trials of remyelinating drugs. It has further significance for neurologists, who might recognise this benign and incidental phenomenon and prevent potentially unnecessary over investigation.
